# Supplementary material for: Single-cell herpes simplex virus type 1 infection of neurons using drop-based microfluidics reveals heterogeneous replication kinetics
Source: Sci Adv. 2024 Feb 28;10(9):eadk9185. doi: 10.1126/sciadv.adk9185 (PMC10901367; doi:10.1126/sciadv.adk9185)
Supplement: Supplementary file 1 — Fig. S1 Legend for movie S1 [file sciadv.adk9185_sm.pdf]

Science Advances
AAAS

Supplementary Materials for

Single-cell herpes simplex virus type 1 infection of neurons using drop-based microfluidics reveals heterogeneous replication kinetics

Jacob P. Fredrikson *et al.*

Corresponding author: Matthew P. Taylor, mptaylor@montana.edu; Connie B. Chang, chang.connie@mayo.edu

Sci. Adv. **10**, eadk9185 (2024)
DOI: 10.1126/sciadv.adk9185

The PDF file includes:

Fig. S1
Legend for movie S1

Other Supplementary Material for this manuscript includes the following:

Movie S1

Fig. S1.

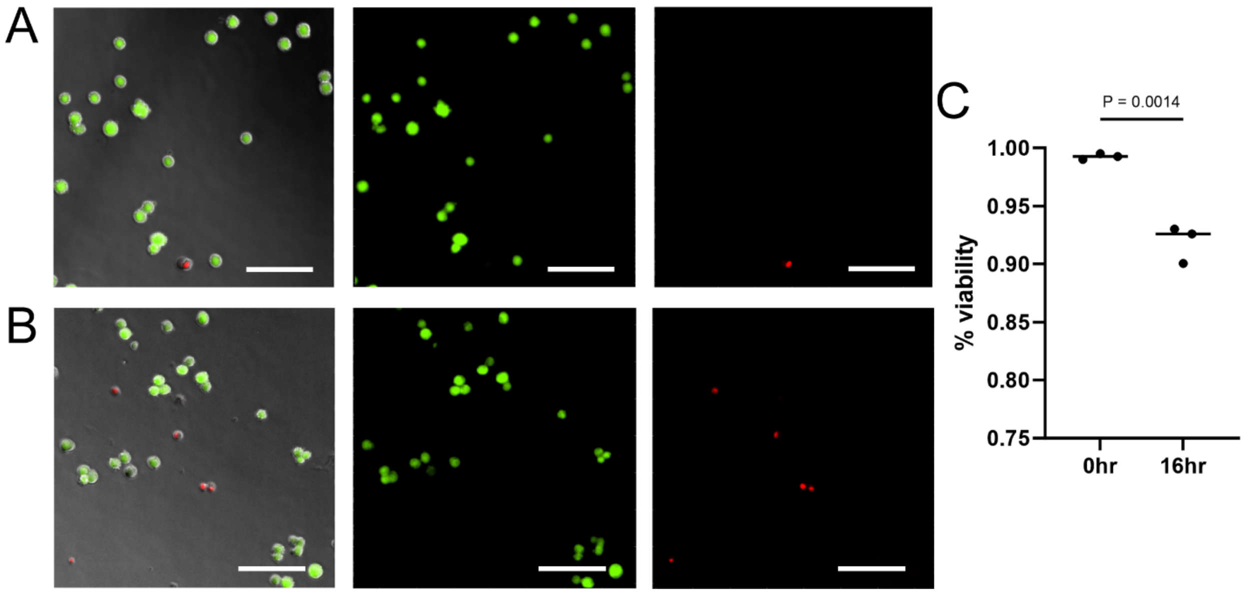

SI Figure 1 - Vero cells cultured in-suspension are viable. Uninfected Vero cells were emulsified and incubated for 16 hours. (A) Cells stained for initial viability at $t = 0$ h and (B) after 16 h of incubation in-suspension. Scale bar is 100 µm in all panels. (C) Viability was quantified by detection of CellTracker Green (GFP) or propidium iodide-stained cells (RFP). $N > 113$ cells per replicate, total $N = 1224$ cells counted. Triplicate cultures were emulsified, stained, and counted. Error represented as SD, statistical comparison performed by unpaired t-test.

Movie S1.

Time-lapse microscopy of an infected neuron expressing YFP and RFP in a DropSOAC chamber (*31*). Single channel images are presented for brightfield, YFP, and RFP, along with a 3 channel merged image. Scale bar = 50 µm. Fluorescent images were acquired every 15 minutes. Movie playback at 5 frames per second.
